# Retraction: New synthesis of 2-aroylbenzothiazoles *via* metal-free domino transformations of anilines, acetophenones, and elemental sulfur

**DOI:** 10.1039/d3ra90023a

**Published:** 2023-03-23

**Authors:** Tien V. Huynh, Khang V. Doan, Ngoc T. K. Luong, Duyen T. P. Nguyen, Son H. Doan, Tung T. Nguyen, Nam T. S. Phan

**Affiliations:** a Faculty of Chemical Engineering, Ho Chi Minh City University of Technology (HCMUT) 268 Ly Thuong Kiet, District 10 Ho Chi Minh City Vietnam tungtn@hcmut.edu.vn ptsnam@hcmut.edu.vn; b Vietnam National University Ho Chi Minh City Linh Trung Ward, Thu Duc District Ho Chi Minh City Vietnam; c Faculty of Chemical Technology, Ho Chi Minh City University of Food Industry (HUFI) 140 Le Trong Tan, Tan Phu District Ho Chi Minh City Vietnam

## Abstract

Retraction of ‘New synthesis of 2-aroylbenzothiazoles *via* metal-free domino transformations of anilines, acetophenones, and elemental sulfur’ by Tien V. Huynh *et al.*, *RSC Adv.*, 2020, **10**, 18423–18433, https://doi.org/10.1039/D0RA01750G.

Tien V. Huynh, Ngoc T. K. Luong, Duyen T. P. Nguyen, Tung T. Nguyen and Nam T. S. Phan hereby wholly retract this *RSC Advances* article due to irreproducibility of the reaction yields.

Khang V. Doan and Son H. Doan were informed about the retraction of the article, but have not responded to any correspondence regarding the retraction.

The results shown in Table 2 (the synthesis of compounds **3aa–3df**) could not be reproduced when freshly opened reagents and solvents were used. It could be reasoned that the reported reagents were contaminated with unknown impurities, which have facilitated the reaction.

The corresponding authors regret this carelessness and apologise for any inconvenience to readers.

Signed: Tien V. Huynh, Khang V. Doan, Ngoc T. K. Luong, Duyen T. P. Nguyen, Son H. Doan, Tung T. Nguyen and Nam T. S. Phan.

Date: 16th March 2023

Retraction endorsed by Laura Fisher, Executive Editor, *RSC Advances*

## Supplementary Material

